# Factors associated with parental intentions to vaccinate 0-4-year-old children against COVID-19 in Canada: a cross-sectional study using the Childhood COVID-19 Immunization Coverage Survey (CCICS)

**DOI:** 10.1186/s12889-024-20874-2

**Published:** 2024-12-18

**Authors:** Israa Zareef, Anna-Maria Frescura, Sailly Dave, Caren Uhlik, David Guan, Hussein Samhat, Marwa Ebrahim, Julie Laroche

**Affiliations:** Vaccine Coverage and Effectiveness Surveillance Division, Infectious Diseases and Vaccination Programs Branch, Public Health Agency of Canada, Ottawa, ON Canada

**Keywords:** Canada, Children, COVID-19, Immunization, Vaccination

## Abstract

**Background:**

The objective of this study was to determine the factors associated with low or no parental intention to vaccinate children of 0-4-years in Canada with a COVID-19 vaccine through the 2022 Childhood COVID-19 Immunization Coverage Survey (CCICS). The CCICS was conducted prior to the introduction of a COVID-19 vaccine and a vaccine recommendation for this age group.

**Methods:**

Simple and multiple logistic regression models were used to determine associations between sociodemographic factors as well as knowledge, attitudes and beliefs and low/no intentions to vaccinate against COVID-19 among parents of children 0–4 years.

**Results:**

Factors associated with low intentions to vaccinate children against COVID-19 included being male (aOR: 2.0; 95% CI: 2.0‒2.1) compared to female; being 30–39 (aOR 1.1; 95% CI: 1.1‒1.2) compared to 40+; being Black (aOR: 2.3, 95% CI: 2.2‒2.5), East/Southeast Asian (aOR: 3.6, 95% CI: 3.3‒3.8), or having multiple ethnicities (aOR: 1.3, 95% CI: 1.1‒1.6) compared to White European ethnicity; living in a rural (aOR: 2.0, 95% CI: 1.9‒2.1) compared to urban community; having a total 2021 household income of $60,000‒$79,999 CAD (aOR: 1.4, 95% CI: 1.3‒1.5) compared to $150,000 CAD and above; and trusting government bodies (aOR: 2.4; 95% CI: 1.1‒1.2), international bodies (aOR: 2.4; 95% CI: 2.2‒2.5), or media (aOR: 2.0, 95% CI: 1.9‒2.2) for information about COVID-19 vaccines compared to health care providers.

**Conclusions:**

The findings of this study demonstrate that several sociodemographic factors and parental beliefs impact the decision to vaccinate children 0–4 years of age against COVID-19. Future research should focus on sociodemographic barriers to vaccination and how to most appropriately tailor the delivery of vaccination programs to specific groups, in an effort to narrow the gap between intentions and uptake of COVID-19 vaccination in younger children. As well, messaging should specifically be targeted to parents who have lower confidence in the COVID-19 vaccine and the government to provide correct information and build trust.

**Supplementary Information:**

The online version contains supplementary material available at 10.1186/s12889-024-20874-2.

## Abstract (significance)

What is already known about this subject?

What does this study add?

How might this impact on clinical practice?

There is limited Canadian research that examines which factors are associated with parental intentions to vaccinate children against COVID-19. Based on a review of studies conducted outside of Canada, some of the factors associated with a higher parental intention to vaccinate their child against COVID-19, include race, income, and vaccine hesitancy.

This study provides information in the Canadian context so that decision makers in Canada can examine which groups of people have a tendency to have lower intentions to vaccinate. This is important because it can help to identify population groups where additional efforts are needed to increasing vaccination coverage rates and identify inequities in program delivery.

In clinical practice, this study can impact how physicians and other healthcare professionals interact with their patients. Healthcare providers are one of the main sources of vaccine information that is passed on to patients. Therefore, understanding which groups of people have lower intentions to vaccinate and which factors are associated with lower intentions to vaccinate can guide healthcare providers to providing even more knowledge where it is most needed.

**Supplementary Information:**

The online version contains supplementary material available at 10.1186/s12889-024-20874-2.

## Introduction

The COVID-19 pandemic has been a source of significant morbidity and mortality worldwide [1]. It has also influenced the way individuals behave and perceive the world [2]. There are differing opinions and views on COVID-19 vaccination and specifically, childhood vaccination against COVID-19 [3]. The COVID-19 vaccine emerged at a time when there was new and at times potentially misleading information in the media and other online platforms regarding vaccines, which may have contributed to hesitancy towards the COVID-19 vaccine among some parents [3]. Furthermore, rapid vaccine development may have also led to safety concerns around the COVID-19 vaccine [3]. COVID-19 vaccine uptake in children remains quite low. As of June 2024, only 8.4% of children 0–4 years in Canada had received at least one dose of a COVID-19 vaccine [4]. However, it is important to note that the COVID-19 vaccine was never heavily targeted towards this age group, unless they were at high risk of severe illness due to COVID-19.

Most children infected with COVID-19 have mild or no symptoms, but some experience hospitalization, long-term effects, or Multisystem Inflammatory Syndrome in children (MIS-C) [5]. COVID-19 vaccines are used to protect children from the negative effects of COVID-19. Although COVID-19 vaccination was rapidly introduced for adults in December 2020, it was not until July 2022 that Health Canada approved a COVID-19 vaccine for children 6 months to 4 years [6]. As of October 2023, the National Advisory Committee on Immunization (NACI) in Canada recommends that children 6 months to under 5 years who are unvaccinated should be vaccinated against COVID-19 if they are at high risk of severe illness, and receive an additional dose of a vaccine if they are moderately to severely immunocompromised [5]. In addition, NACI provided a discretionary recommendation to vaccinate children 6 months to under 5 years of age who are previously unvaccinated against COVID-19 and are not at high risk of severe illness due to COVID-19 [5]. A publicly-funded program for COVID-19 vaccine delivery was rolled out in all Canadian provinces and territories by December 2020. Determining factors associated with parental intentions to vaccinate children against COVID-19 is necessary to support decision-making processes and highlight concerns parents may have prior to or even after vaccine introduction.

Previous literature has found several factors associated with intentions to vaccinate children against COVID-19, but few have been conducted in Canada. A prospective cohort study conducted in the United States (US) found that safety and efficacy of the COVID-19 vaccine, as well as lack of trust in the government led to lower parental intentions to vaccinate children 4 months to 4 years [7]. A descriptive correlational study conducted in the US examined factors associated with parental intentions to vaccinate against COVID-19 and compared these factors to those related to intentions to vaccinate against influenza [8]. This study also found that parents’ views on the safety and effectiveness of the COVID-19 vaccine impacted their intentions to vaccinate [8]. Another US study comparing vaccine-related attitudes and values of parents of children 2–17 years found that the most prevalent concerns about the COVID-19 vaccine was the speed of development, side effects, and suspicion of the government [9]. One study conducted in Latin America and the Caribbean found that parents older than 35 years of age, educational level above college, and having a chronic medical condition were associated with higher parental intentions to vaccinate their children and adolescents against COVID-19 [10]. They also found that parents living in rural areas had lower intentions to vaccinate their children [10]. A systematic review conducted in Canada found that females, individuals living in rural areas, non-White individuals, and those with a high school education or less were associated with higher levels of vaccine hesitancy and unwillingness to vaccinate [11].

To bridge the gap in the literature available in the Canadian context, this study examined 2022 Childhood COVID-19 Immunization Coverage Survey (CCICS) data to determine the proportion of parents in Canada who did and did not intend to vaccinate their child against COVID-19 once their child was eligible for the vaccine. In addition, this study investigated the factors associated with parental intentions to vaccinate children 0–4 years who were not yet eligible to receive a COVID-19 vaccine at the time of data collection for the 2022 CCICS.

## Methodology

### Study design and data source

The data source used to conduct this analysis was the 2022 Childhood COVID-19 Immunization Coverage Survey (renamed to “Childhood Seasonal Immunization Coverage Survey” in 2024) [12]. CCICS is an annual vaccine coverage survey conducted by the Public Health Agency of Canada (PHAC) to estimate COVID-19 and influenza vaccine coverage among children less than 18 years of age, as well as parental knowledge, attitudes and beliefs towards these vaccines. The CCICS also collects information on parental intentions to vaccinate children that were not eligible for vaccination or were not vaccinated, and the barriers or facilitators associated with COVID-19 and influenza childhood vaccination.

Participants were recruited from a general population sample by random digit dialing. A total of 10,536 parents/guardians living in Canada and aged 18 years or older were surveyed using Web surveys or computer assisted telephone interviewing. Data collection occurred between April 7 and July 21, 2022. Results of the survey were weighted by the child’s age, sex at birth, and province of residence. At the time of data collection, COVID-19 vaccines for children under 5 years of age were not approved. Therefore, this cycle of the CCICS did not include vaccine coverage estimation for this age group, but rather intention to vaccinate these children in the future, once they become eligible.

### Participants

The study population for this analysis was limited to parents/guardians of children 0–4 years. The total sample size for this analysis was 2,316.

### Variables and measurements

The outcome variable in this analysis was parental intention to vaccinate 0-4-year-old children against COVID-19. In the CCICS, parents with children 0–4 years were asked, “In the future, if your child becomes eligible for the COVID-19 vaccine, how likely is it that you will get your child vaccinated?” The response options were “Definitely will,” “Probably will,” “Probably won’t,” “Definitely won’t,” “Don’t know,” and “Prefer not to answer.” Participants who answered “Don’t know” or “Prefer not to answer” were excluded (*n* = 192). To create a binary variable, “Definitely will” and “Probably will” responses were combined to create a “high intention” category and “Definitely won’t” and “Probably won’t” responses were combined to create a “low intention” category.

Predictor variables were included in the analysis based on factors previously established in the literature associated with parental intentions to vaccinate children against COVID-19 [7–11]. The sociodemographic variables included in the analysis were parent’s sex, age, ethnicity, urban/rural status, education level, and household income. Due to small sample sizes, both the Latin American and the Indigenous from another part of the world groups were included in the “Other” category of the ethnicity variable. Additional predictor variables included were: whether the child had a medical condition and the most trusted sources of information used by parents regarding COVID-19 vaccines. Finally, a vaccine-confidence score was created using KAB statements. The vaccine-confidence score was a continuous variable that was derived by combining four general or COVID-19 vaccine KAB statements [13]. For each of the KAB statements, parents could respond “Strongly agree,” “Somewhat agree,” “Somewhat disagree,” “Strongly disagree,” “Don’t know,” or “Prefer not to answer.” “Prefer not to answer” responses were not included. A composite score representing the average parental vaccine confidence towards these statements was created, such that strongly agree = 5, somewhat agree = 4, don’t know = 3, somewhat disagree = 2, and strongly disagree = 1.^13^ A score of 1 was a very low confidence in vaccines, 2 was a low confidence in vaccines, 3 was don’t know, 4 was a high confidence in vaccines, and 5 was a very high confidence in vaccines. A continuous variable was then created by deriving a mean score for the four KAB statements, where the higher the mean score, the higher the parental vaccine confidence [13]. More information about the vaccine-confidence variable can be found in Appendix A of the Supplementary Material.

### Statistical analysis

Data analysis was conducted with SAS Enterprise Guide 7.1. The proportion of missing/unknown data (i.e., “don’t know” or “prefer not to answer”) was estimated for all predictor variables. Majority of the variables had missing data that did not account for more than 10% of the sample data, therefore these missing data categories were not included in the analysis [14, 15]. The variable with the highest proportion of missing data was the income variable, with 5% of parents having unknown (“don’t know” and “prefer not to answer”) income. A chi-squared test of association between parental intention and the “unknown” category of the income variable was significant and thus, the unknown category for income was included as a valid response. The overall percentage of participants excluded from the 2,542 total respondents due to missing data was 9%. Therefore, the total analytical sample consisted of 2,316 parents. Since missing data was less than 10% and the missing data was missing at random after performing Little’s MCAR test, complete case analysis was conducted [14]. A supplemental analysis on the missing data was done on all incomplete cases to determine if there was a significant difference between the analytical sample and the sample with missing data (Appendix B, Supplementary Material). Unweighted frequencies were calculated to ensure adequate cell size counts for analysis and regrouping of response options was done if the cell counts were too low. The mean, median, and standard deviation (SD) for the vaccine-confidence score was also calculated as part of the descriptive statistics. Weighted frequencies with 95% confidence intervals (CIs) were also calculated. To account for complex survey design and weight adjustments, 500 bootstrap weights were applied to determine weighted proportions and odds ratios as well as weighted confidence intervals.

Simple logistic regression models of low parental intent against each predictor variable were developed to determine the association between each predictor variable and the outcome variable (low intent), yielding unadjusted odds ratios (ORs). Multiple regression analysis was conducted, yielding adjusted odds ratios (aORs) and 95% CIs. Predictors were included in the final logistic regression model based on factors previously established in the literature, as well as significance in the simple regression models, only if deemed necessary. As well, variables were removed from the model if they were correlated with other variables, creating collinearity issues. Linearity of the vaccine-confidence score was assessed by generating and observing the scatter plot between the vaccine-confidence score and the logit values of parental intent. Collinearity was assessed by running Chi-square tests between categorical variables to determine if they differ or not. For example, a Chi-squared test of employment status with education and income showed that these variables were highly correlated, therefore employment status was removed because income and education levels provided similar information that could be used to interpret the study findings. Parental hesitancy variable was also not included in the model because it was highly correlated with the vaccine-confidence score, but it was retained in the descriptive results to provide relevant background on the study population. If two variables were correlated and no other variable in the model could provide the same insight if one of the correlated variables was omitted, both variables were retained in the model. This was done if inflated standard errors or other issues with the model were not observed.

### Ethical considerations & consent

The CCICS was approved by the Health Canada Public Health Agency of Canada Research Ethics Board and informed consent was obtained from respondents of the survey [16]. 

## Results

### Sample characteristics

Approximately 65% (*n* = 1676) of parents with children 0–4 years of age had higher intentions to vaccinate their child against COVID-19 once the vaccine becomes available, while 35% (*n* = 886) of parents had low or no intention to vaccinate their child in the future (*p* < 0.0001). The majority of parents of 0–4 year olds were between the ages of 30–39 (64%), self-reported as White European (76%), lived in an urban community setting (85%), and had a total household income of $150,000 CAD or more (36%) (Table 1). There was a generally even split between the three education levels of parents; below bachelor’s (35%), bachelor’s (33%), and above bachelor’s (31%). Furthermore, 45% of parents reported being hesitant to vaccinate their child against COVID-19. A chronic medical condition was reported among 8% of children. Finally, 44% of parents reported that healthcare providers were their most trusted source of information regarding COVID-19 vaccines, while 27% trusted government bodies, and 5% trusted media sources, such as news or social media. This sample of parents had a mean vaccine-confidence score of 4 ± 0.01 (high confidence).

**Table 1:** Demographic characteristics of parents of 0-4-year-olds overall and stratified by intention to vaccinate child against COVID-19

	Overall, *N* (Weighted %)	Definitely/probably will not vaccinate, *N* (Weighted %)	Definitely/probably will vaccinate, *N* (Weighted %)
**Parent’s Sex Assigned at Birth**			
Female	1539 (60.7)	441 (50.2)	1098 (66.4)
Male	990 (38.8)	415 (48.6)	575 (33.4)
**Child’s Sex Assigned at Birth**			
Male	1321 (51.7)	432 (49.8)	889 (52.7)
emale	1221 (48.3)	434 (50.2)	787 (47.3)
**Parent’s Age**			
40+	741 (29.8)	215 (24.5)	526 (32.7)
30–39	1610 (63.9)	547 (64.0)	1063 (63.8)
18–29	173 (5.5)	90 (9.6)	83 (3.28)
**Parent’s Race/Ethnicity**			
White European	1945 (76.2)	634 (72.6)	1311 (78.1)
Black	73 (3.2)	32 (4.2)	41 (2.6)
East/Southeast Asian	99 (3.6)	23 (2.7)	76 (4.1)
South Asian	81 (3.5)	16 (2.0)	65 (4.4)
Middle Eastern and North African	49 (2.1)	17 (2.1)	32 (2.2)
Indigenous	42 (0.9)	16 (1.2)	26 (0.68)
Other	91 (3.9)	39 (4.8)	52 (3.4)
Multiple Ethnicities^*^	75 (3.0)	26 (2.9)	49 (3.1)
**Community Type** ^**^			
Urban	2053 (84.8)	639 (76.6)	1414 (89.3)
Rural	461 (14.1)	207 (21.1)	254 (10.3)
**Parent’s Education Level**			
Above bachelor’s	938 (34.8)	460 (52.2)	478 (25.3)
Bachelor’s	834 (32.9)	212 (25.0)	622 (37.3)
Below bachelor’s	745 (31.2)	176 (20.8)	569 (36.9)
**Parent’s Income**			
$150,000 and above	868 (36.0)	213 (25.7)	655 (41.6)
$100,000-$149,999	677 (27.5)	235 (27.8)	442 (27.3)
$80,000-$99,999	289 (10.7)	112 (12.7)	177 (9.6)
$60,000-$79,999	230 (8.5)	102 (11.6)	128 (6.8)
Under $60,000	347 (12.2)	144 (14.9)	203 (10.7)
Unknown	131 (5.2)	60 (7.4)	71 (4.0)
**Chronic medical condition of child** ^**†**^			
No	2307 (91.8)	794 (93.4)	1513 (90.9)
Yes	207 (8.2)	64 (6.6)	153 (9.1)
**Parental COVID-19 vaccine hesitancy** ^**‡**^			
No	1370 (52.9)	100 (10.6)	1270 (76.0)
Yes	1106 (44.5)	738 (86.3)	368 (21.6)
**Most trusted source of information about COVID-19 vaccines**			
Health care providers	1071 (43.5)	260 (30.2)	811 (50.7)
Government bodies	743 (27.2)	106 (11.4)	637 (35.9)
International bodies	158 (5.8)	86 (9.2)	72 (4.0)
Media	123 (4.9)	52 (6.2)	71 (4.1)
Other	236 (10.1)	177 (23.9)	59 (3.6)
Don’t know	102 (4.5)	79 (10.6)	23 (1.5)
**Vaccine-confidence score** ^**§**^			
Mean ± standard deviation	3.7 (± 0.1)	2.0 (± 0.1)	4.6 (± 0.1)

^*^ Multiple ethnicities: included any parent that answered “yes” to more than one race category [12]

^**^ Community type: type of area that parent resides in. An urban area is a city, town or village with a population of 1000 people or more, while a rural area is any other area of lower population [12]

^**†**^ Chronic medical condition of child: included any child that had one or more of the following conditions- sickle cell anemia or thalassemia major, neurologic or neurodevelopmental disorder, asthma or chronic lung disease, chronic liver, heart or kidney disease, diabetes, obesity, or Down syndrome, immune suppression, cancer, or any other medical condition [12]

^**‡**^ Parental COVID-19 vaccine hesitancy: included parents who are or were hesitant to vaccinate their child against COVID-19 [12]

^**§**^ Vaccine-confidence score: continuous variable derived from knowledge, attitude, and belief statements. Scale of 1–5; higher score means more confidence in vaccines [12]

### Sociodemographic factors associated with low or no parental intent

Male parents were more likely to have lower intentions to vaccinate their 0-4-year-old child against COVID-19 as compared to female parents (aOR: 2.0; 95% CI: 2.0–2.1) (Table 2). Younger parents aged 30 to 39 were also more likely to have lower intentions to vaccinate their child against COVID-19 (aOR: 1.1, 95% CI:1.1‒1.2) when compared to older parents aged 40 years and above. Other factors associated with lower intentions to vaccinate 0-4-year-old children against COVID-19 included parents self-identifying as Black (aOR: 2.3; 95% CI:2.2‒2.5), East/Southeast Asian (aOR: 3.6; 95% CI: 3.3‒3.8), or having multiple ethnicities (aOR: 1.3; 95% CI: 1.1‒1.6) compared to White European ethnicity; and living in a rural community (aOR: 2.0; 95% CI: 1.9‒2.1) compared to urban community. Parents with a household income of $60,000-$79,999 CAD were more likely to have lower intentions to vaccinate their child (aOR: 1.4 (95% CI: 1.3‒1.5) compared to parents with a higher household income of $150,000 CAD and above.

Lower intentions to vaccinate were also seen in parents whose most trusted source of information regarding COVID-19 vaccines was government bodies (aOR:1.2; 95% CI: 1.1‒1.2), international bodies (aOR: 2.4; 95% CI: 2.2‒2.5) or media, such as news or social media (aOR: 2.0; 95% CI: 1.9‒2.2) when all were compared to health care providers. In addition, parents who responded that they didn’t know who their most trusted source of information about COVID-19 vaccines was also had lower intentions to vaccinate their child as compared to those who trusted healthcare providers (aOR: 1.3; 95% CI: 1.2‒1.4).

**Table 2:** Unadjusted and adjusted odds ratios of low parental intent to vaccinate 0-4-year-olds against COVID-19

	Unadjusted OR (95% CI)	Adjusted OR (95% CI)
**Parent’s Sex Assigned at Birth**		
Female	Reference	Reference
Male	1.9 (1.9, 2.0)	**2.0 (2.0**,** 2.1)**
**Parent’s Age**		
40+	Reference	Reference
30–39	1.3 (1.3, 1.4)	**1.1 (1.1**,** 1.2)**
18–29	3.9 (3.8, 4.1)	1.0 (1.0, 1.1)
**Parent’s Race/Ethnicity**		
White European	Reference	Reference
Black	1.7 (1.6, 1.8)	**2.3 (2.2**,** 2.5)**
East/Southeast Asian	0.7 (0.7, 0.7)	**3.6 (3.3**,** 3.8)**
South Asian	0.5 (0.5, 0.5)	0.9 (0.9, 1.0)
Middle Eastern and North African	1.0 (1.0, 1.1)	1.1 (1.0, 1.2)
Indigenous	2.0 (1.7, 2.2)	1.0 (0.8, 1.2)
Other	1.5 (1.5, 1.6)	1.0 (0.9, 1.0)
Multiple Ethnicities^*^	1.0 (1.0, 1.0)	**1.3 (1.1**,** 1.6)**
**Community Type** ^**^		
Urban	Reference	Reference
Rural	2.4 (2.3, 2.4)	**2.0 (1.9**,** 2.1)**
**Parent’s Education Level**		
Above bachelor’s	Reference	Reference
Bachelor’s	1.2 (1.2, 1.2)	**0.5 (0.5**,** 0.5)**
Below bachelor’s	3.7 (3.6, 3.7)	**0.7 (0.7**,** 0.8)**
**Parent’s Income**		
$150,000 and above	Reference	Reference
$100,000-$149,999	1.6 (1.6, 1.7)	1.0 (0.9, 1.0)
$80,000-$99,999	2.1 (2.1, 2.2)	1.1 (1.0, 1.1)
$60,000 -$79,999	2.7 (2.7, 2.8)	**1.4 (1.3**,** 1.5)**
Under $60,000	2.3 (2.2, 2.3)	1.1 (1.0, 1.1)
Unknown	3.0 (2.9, 3.1)	**0.5 (0.5**,** 0.6)**
**Medical condition of child** ^**†**^		
No	Reference	Reference
Yes	0.7 (0.7, 0.7)	**0.8 (0.8**,** 0.8)**
**Most trusted source of information about COVID-19 vaccines**		
Health care providers	Reference	Reference
Government bodies	0.5 (0.5, 0.5)	**1.2 (1.1**,** 1.2)**
International bodies	3.9 (3.7, 4.0)	**2.4 (2.2**,** 2.5)**
Media	2.5 (2.4, 2.6)	**2.0 (1.9**,** 2.2)**
Other	9.8 (9.5, 10.1)	1.1 (1.0, 1.1)
Don’t know	10.0 (9.6, 10.5)	**1.3 (1.2**,** 1.4)**
**Vaccine-confidence score** ^**§**^	0.04 (0.04, 0.04)	**0.03 (0.03**,** 0.04)**

Note: statistically significant aORs are bolded

^*^ Multiple ethnicities: included any parent that answered “yes” to more than one race category [12].

^**^ Community type: type of area that parent resides in. An urban area is a city, town or village with a population of 1000 people or more, while a rural area is any other area of lower population [12]

^**†**^ Chronic medical condition of child: included any child that had one or more of the following conditions- sickle cell anemia or thalassemia major, neurologic or neurodevelopmental disorder, asthma or chronic lung disease, chronic liver, heart or kidney disease, diabetes, obesity, or Down syndrome, immune suppression, cancer, or any other medical condition [12]

^**§**^ Vaccine-confidence score: continuous variable derived from knowledge, attitude, and belief statements. Scale of 1–5; higher score means more confidence in vaccines [12]

Parents were less likely to have lower intentions to vaccinate their child if they had bachelor’s (aOR: 0.5; 95% CI:0.5‒0.5) or below bachelor’s education (aOR: 0.7; 95% CI: 0.7‒0.8) compared to above bachelor’s education level; if they had an unknown (responded “prefer not to answer” or don’t know) income (aOR: 0.5; 95% CI: 0.5‒0.6) compared to $150,000 CAD and above; and if they reported their child as having a medical condition (aOR: 0.8; 95% CI: 0.8‒0.8) compared to those without one. Having higher vaccine confidence was also associated with being less likely to have lower intentions to vaccinate their child against COVID-19 (aOR: 0.03; 95% CI: 0.03‒0.04).

## Discussion

Results from this analysis showed that approximately 65% of parents with children 0–4 years of age intended to vaccinate their child against COVID-19 prior to the vaccine becoming available. This proportion is considerably higher than recent COVID-19 coverage for this age group, which is 8.4% for children who have had at least one dose as of June 2024 [4]. At the time the CCICS was conducted, the COVID-19 vaccine was only approved for children older than 4 years of age and NACI strongly recommended that children 5 years and older should be vaccinated with a COVID-19 vaccine if there were no contraindications to the vaccine [17]. It is important to note that once NACI released a statement regarding COVID-19 vaccination in 0-4-year-olds, it was a discretionary recommendation, not a strong one, and more emphasis was placed on children at risk of moderate to severe infection from COVID-19 [5]. Nevertheless, parental intentions might not be an accurate indicator of future vaccine uptake for this population. These results align with previous research on the discrepancy between intentions to vaccinate and actual coverage rates for this age group, which is known as the intention-action gap [18]. A similar study examining the relationship between H1N1 vaccination intentions and vaccination coverage found similar findings, where there was also a large discrepancy between the intention to vaccinate and coverage rates [19]. This study suggests that considering the barriers and reasons for low intentions to vaccinate could mitigate this discrepancy and potentially improve vaccine coverage rates in the future [19]. 

There were several results of this study that were consistent with the literature on factors affecting low intentions to vaccinate against COVID-19 [7, 8, 10, 11, 20–25]. It is important to establish these factors because it can help organizations tailor their communications towards specific sub-populations with anticipated lower coverage. Similar to previous studies, younger parents were more likely to have lower intentions to vaccinate their children than older parents [20]. A similar study by Humble et al. examining parental intentions to vaccinate children 0–17 years of age in Canada also found that the older the parent, the higher their intention to vaccinate [21]. Some of the reasons for this is could be that younger people are more likely to use social media, where false and negative information was heavily present around the COVID-19 pandemic [20]. Another potential reason for this association is that COVID-19 is more severe in older adults as compared to younger adults, which might lead older parents to vaccinate their children more often to avoid potentially severe outcomes for their children as well [22]. 

Our study shows that Black parents and also parents with lower income levels were more likely to have low intentions to vaccinate their child against COVID-19, which is consistent with the literature [10, 23]. Humble et al. found that parents who reported a household income level of less than $80,000 CAD had lower intentions to vaccinate their children against COVID-19 from their unadjusted models, but once models were adjusted, the odds ratios were non-significant [21]. They determined that other factors, such as employment part-time compared to full-time, children not receiving flu vaccine before the pandemic, and believing COVID-19 vaccine is not necessary or unsafe was associated with low intentions to vaccinate against COVID-19 [21]. In our analysis, we did not include employment type or flu vaccine status. This could pose as a limitation of our research since these variables were present in the CCICS, but we did not choose to include them due to either collinearity or inclusion of other variables that captured similar points. Some barriers that might be leading to the association between income level and ethnicity with lower intentions are fears of discrimination or racism, transportation issues, community attitudes and beliefs, and lack of trust in the government [13]. 

This study also demonstrated that a higher vaccine confidence is associated with being less likely to have lower intentions to vaccinate children against COVID-19. Possible reasons for decreased confidence towards vaccines could be lower levels of education and lower income as well as visible minority status [24]. 

This study emphasizes the need to address vaccine hesitancy among parents who belong to racial minority groups, have lower income, and are younger. As mentioned previously, barriers to vaccine uptake and intention in these populations could be due to lack of accessibility, community attitudes and beliefs, and lack of trust in the government [13]. Increasing vaccine confidence, though challenging, could be accomplished by increasing vaccine knowledge and awareness in marginalized communities, improving access and convenience of vaccination, and engaging and communicating with lower vaccine uptake communities to address mistrust in the government [25]. Furthermore, healthcare providers, in their interactions with patients, have the ability to enhance knowledge on vaccines [26]. This includes sharing side effects of vaccination, vaccine safety, addressing struggles faced by patients, and overall building trust and open communication with patients [26]. Fostering a healthy and trustful relationship between healthcare providers and patients enables patients to access correct information about vaccines, rather than misinformation from other sources, such as media [26]. 

The following limitations should be taken into consideration when interpreting the findings from this study. First, the data collected in the 2022 CCICS was self-reported which has the potential to result in recall bias or social desirability bias. Participants of the survey might not have remembered exact details from the past and they might have reported information in a manner to be more favourably viewed by others, which are common phenomena in self-reported data. Second, the cross-sectional nature of the CCICS means that causality cannot be established between factors affecting intentions. Finally, a portion of the sample was excluded from the analysis because of incomplete data. Specifically, 9% of the total sample contained incomplete data. Analysis on the excluded participants highlighted demographic differences between this sample and the analytical sample, including parental sex, age, ethnicity, community type, and education (see Appendix B, Supplementary Material). However, the proportion of missing data was less than the typical 10% threshold in which missing data becomes a greater issue [14]. Nevertheless, our findings need to be interpreted with caution since excluded participants could have distinct characteristics or behaviours that differed from the analytical sample. Thus, the results are less generalizable. Despite these limitations, the main strength of this study is that it is a large nationally representative sample, representing approximately 7 million children younger than 18 years of age, across Canada. Furthermore, it allows us to understand parents’ perceptions and intentions to vaccinate their children at the height of the COVID-19 pandemic.

## Conclusion

This study emphasizes the importance of addressing sociodemographic factors and knowledge, attitudes, and beliefs related to the COVID-19 vaccine in future messaging and vaccine promotion campaigns. It has also established the need to focus vaccine messaging in minority communities, lower income communities, and younger parents since these factors were associated with lower intentions to vaccinate children. Additional research should focus on the specific barriers that these parents face that might lead them to having low intention or low vaccine uptake. Future CCICS cycles will capture COVID-19 vaccine status for 0-4-year-olds and be able to provide a good comparison between intention from previous survey cycles and uptake in future cycles. This, combined with the inclusion of more research on barriers affecting vaccine intention/uptake in this age group, can lead to a greater understanding of what is needed to improve vaccine uptake for this subset of the population.

## Electronic supplementary material

Below is the link to the electronic supplementary material.

Supplementary Material 1

## Data Availability

To protect the confidentiality of survey respondents, the full survey dataset cannot be released. All data generated during this study is included in this published article.
