# Supplementary material for: Factors associated with parental intentions to vaccinate 0-4-year-old children against COVID-19 in Canada: a cross-sectional study using the Childhood COVID-19 Immunization Coverage Survey (CCICS)
Source: BMC Public Health. 2024 Dec 18;24:3456. doi: 10.1186/s12889-024-20874-2 (PMC11658274; doi:10.1186/s12889-024-20874-2)
Supplement: Supplementary file 1 — Supplementary Material 1 [file 12889_2024_20874_MOESM1_ESM.docx]

**Supplementary Material**

**Appendix A. Vaccine-confidence variable derivation**

4 KAB statements were deemed relevant to the research question based on evidence from the literature and the specific research question of interest. KAB statements regarding the flu were removed since this analysis focused on COVID-19. KAB statement regarding public health measures was removed because these were no longer in place.

The statements that were included were:

1. In general, COVID-19 vaccines are safe.

2. In general, COVID-19 vaccines are effective.

3. Vaccination is a safer way to build immunity against COVID-19 than getting

infected.

4. My child needs to be vaccinated against COVID-19 even after infection.

There were five possible response options to the KAB statements that we scored from 1-5: strongly disagree (1), somewhat disagree(2), don’t know (3), somewhat agree (4), and strongly agree (5). We created a composite score by taking the average of the 5 statements combined. It made sense to do this since all the statements were positive and not a mix of positive and negative statements. Each respondent was assigned a vaccine-confidence score that ranged from 1 to 5. For example, Person A responded somewhat disagree to all KAB statements so (2 score x 5 questions)/5=2 average score for Person A. We did this for all participants and then took the mean. The composite score ranged between 1 and 5. The higher the score, the higher the confidence in vaccines.

**Appendix B**

**Table B.** Differences in demographic variables between the analytic samples and sample with missing data.

|  | **Analytical sample (n=2542)** | **Sample with missing data (n=226)** |  |
| --- | --- | --- | --- |
|  |  |  |  |
| **Key demographic variables** | **Unweighted n (weighted %)** | **Unweighted n (weighted %)** | **Rao-Scott likelihood ratio chi-square test** |
| **Parent’s Sex assigned at birth** |  |  |  |
| Male | 886 (38.0) | 104 (49.0) | p<0.0001 |
| Female | 1430 (62.0) | 109 (51.0) |  |
| **Parent’s Age** |  |  |  |
| 40+ | 678 (30.1) | 63 (29.1) | p<0.0001 |
| 30-39 | 1492 (65.0) | 118 (57.7) |  |
| 18-29 | 146 (4.8) | 27 (13.1) |  |
| **Parent’s Race/Ethnicity** |  |  | p<0.0001 |
| White European | 1837 (79.1) | 108 (78.6) |  |
| Black | 68 (3.3) | F (3.4) |  |
| East/Southeast Asian | 91 (3.7) | F (4.6) |  |
| South Asian | 77 (3.7) | F (3.1) |  |
| Middle Eastern and North  African | 48 (2.3) | F (0.9) |  |
| Indigenous | 39 (0.9) | F (1.0) |  |
| Other | 86 (4.0) | F (4.1) |  |
| Multiple Ethnicities^*^ | 70 (3.0) | F (4.4) |  |
| **Community Type^**^** |  |  |  |
| Urban | 1911 (86.7) | 142 (74.7) | p<0.0001 |
| Rural | 405 (13.3) | 56 (25.3) |  |
| **Parent’s Education Level** |  |  |  |
| Below bachelor’s | 820 (32.9) | 118 (60.2) | p<0.0001 |
| Bachelor’s | 785 (34.2) | 49 (23.1) |  |
| Above Bachelor’s | 711 (32.9) | 34 (16.7) |  |

^*^ Multiple ethnicities: included any parent that answered “yes” to more than one race category.^12^

**^**^** Community type: type of area that parent resides in. An urban area is a city, town or village with a population of 1000 people or more, while a rural area is any other area of lower population.^12^

F Unweighted n cannot be provided due to low cell size count and confidentiality issues.
